# Antioxidants Attenuate Isolation- and L-DOPA-Induced Aggression in Mice

**DOI:** 10.3389/fphar.2017.00945

**Published:** 2018-01-15

**Authors:** Sundas Hira, Uzma Saleem, Fareeha Anwar, Bashir Ahmad

**Affiliations:** ^1^Department of Pharmacy, Riphah Institute of Pharmaceutical Sciences, Riphah International University Lahore, Lahore, Pakistan; ^2^Faculty of Pharmaceutical Sciences, Government College University, Faisalabad, Pakistan

**Keywords:** antioxidant, aggression, L-DOPA, GSH, SOD, CAT

## Abstract

Aggression is a major hallmark worldwide attributing negative traits in personality. Wide variety of antioxidants is used for the treatment of many ailments. The present study was conducted to evaluate the role of antioxidants such as ascorbic acid (15.42 and 30.84 mg/kg), beta carotene (1.02 and 2.05 mg/kg), vitamin E (2.5 and 5.0 mg/kg), and N-acetyl cysteine (102.85 and 205.70 mg/kg) in the treatment of aggression. Two aggression models (isolation induced aggression model and L-DOPA induced aggression model) were used in the study. Male albino mice (*n* = 330) were used in the study which were further subdivided into 11 groups (Group I-control, group II-diseased, group III-standard group, group IV–V treated with ascorbic, group VI–VII treated with beta carotene, group VIII–IX treated with vitamin E, group X–XI treated with N-acetyl cysteine for 14 consecutive days). Different biochemical markers (glutathione, superoxide dismutase, and catalase) were determined to evaluate the antioxidant potential in oxidative stress. High dose of vitamin E (5.0 mg/kg) was more effective to reduce the aggression in isolated animals while all other antioxidants produced dose-dependent anti-aggressive effect except N-acetyl cysteine which had marked anti-aggressive effect at low dose (102.75 mg/kg). Low doses of vitamin E (2.5 mg/kg) and N-acetyl cysteine (102.75 mg/kg) and high dose of beta carotene (2.05 mg/kg) were effective to prevent all aggression parameters in acute anti-aggressive activity against L-DOPA induced aggression. However, all test antioxidants were equally effective in chronic anti-aggressive studies against L-DOPA induced aggression. It may be concluded that selected antioxidants can reverse the aggression which is a key symptom of many neurological disorder.

## Introduction

Brain regions which are involved in regulation of emotions are hypothalamus, prefrontal cortex, amygdala, anterior cingulated cortex, hippocampus, insular cortex, ventral striatum, medial preoptic area, and interconnected structures. Any deformity in the structure or function of these brain regions can cause aggression which is a leading cause of many neurological disorders like mania and schizophrenia. Aggression is the behavior with intention to target any other individual to cause harm (Berkowitz, 1989). The behavior which has enormous or extreme harm is known as violence. Two dominant types of aggression are hostile and instrumental aggression. Hostile aggression is the type of aggression which is not planned and impelled by anger with a purpose to cause harm, as a reaction to some inducement. The other type of aggression is instrumental aggression which is planned with intention to achieve some targets or goals (Anderson and Bushman, 2002).

Many physiological and pathophysiological processes like inflammation, infection, stress, emotional disturbance, and various pharmacological treatments increase the concentration of oxidative molecules such as reactive oxygen species (ROS) and reactive nitrogen species (RNS) (Tsaluchidu et al., 2008). The chemical species which are reactive, possessed the free unpaired electrons in their outer orbits (Valko et al., 2007). Oxidative stress occurs as a result of imbalance between generations of ROS and its detoxification in the body which leads to tissue damage and changes in the defense mechanism (Mittler, 2002). Alterations in defense mechanism cause the depletion of various enzymatic levels like catalase (CAT), Glutathione (GSH), vitamin A and C, etc. (Gutteridge and Halliwell, 2000; Aksenova et al., 2005). Decreased levels of endogenous antioxidants such as [GSH, superoxide dismutase (SOD), and CAT] increase the oxidative stress, associated with the anxiogenic behavior in mice (Masood et al., 2008).

Neurodegenerative changes and ROS lead to decline the level of COMT and MAO-A, resultantly high levels of nor epinephrine and dopamine are responsible for aggression (Volavka et al., 2004). Genetic mutations of noradrenalin receptors specifically ADRB1receptors may have role in aggression and stress. ADRB1 are the type of receptors where β blockers exert their actions. Therefore β receptor blockers have been used to treat aggression (Craig and Halton, 2009).

Antioxidants are the substances which can act as scavengers, remove oxygen, or as precursors. They perform their functions by retarding the formation of ROS and by attaching with the metal ions required for the catalysis of ROS production (Gilgun-Sherki et al., 2002). The study was conducted to evaluate the potential of antioxidants in the management of aggression.

## Materials and Methods

### Drugs and Chemicals

Beta carotene, ascorbic acid, trichloroacetic acid, 5, 5′-dithio-bis-(2-nitrobenzoic acid) (DTNB), pyrogallol, potassium hydroxide, di-potassium hydrogen phosphate, potassium di-hydrogen phosphate were purchased from Sigma–Aldrich (United States). Diazepam was obtained from ROCHE Pharmaceuticals. Vitamin E was obtained from Merck Pharmaceuticals. N-acetyl cysteine was obtained from Abbot Pharmaceuticals. Levodopa and carbidopa were obtained from Hansel Pharmaceuticals (Lahore, Pakistan).

### Experimental Animals

This study was carried out on animals after obtaining permission from research ethical committee of Riphah International University with an authorized number of REC/RIPS-LHR/2017/001 ruled under the regulation of Institute of Laboratory Animal Resources, Commission on Life Sciences University, National Research Council (1996). Male swiss albino mice (*n* = 330) were purchased from Veterinary Research Institute (VRI) Lahore. They were 9 weeks old and ranging in weight 30–40 g at the beginning of the experiment. All mice were housed in animal house of Riphah International University under 12 h light and dark cycles at 22 ± 2°C temperature and 45–55% humidity to acclimatize them. They had free access to food and water *ad libitum.*

### Experimental Protocol

The animals were divided into 11 groups having 10 mice in each group (*n* = 10) for each aggression models. Group I served as negative control group treated with vehicle (1 mL/kg), Group II served as diseased control group treated with (carbidopa 4.28 mg/kg and levodopa 375 mg/kg) or by isolation according to the method of induction. Group III served as standard group treated with diazepam 2.5 mg/kg. Group IV–V treated with ascorbic acid at two dose levels (15.42 and 30.84 mg/kg). Group VI–VII treated with beta carotene at two dose levels (1.02 and 2.05 mg/kg). Group VIII–IX treated with vitamin E at two dose levels (2.5 and 5.0 mg/kg). Group X–XI treated with NAC at two dose levels (102.85 and 205.70 mg/kg).

#### Isolation Induced Aggression

Aggression was induced in each group by isolation method for a period of 1 month. During whole month, each animal was placed in a separate cage (Polypropylene cage 22 cm × 37 cm) with free access to water and food. After 1 month of isolation, aggression behavior of each animal was observed by introducing the intruder in each cage containing the isolated animal (Umukoro et al., 2013). The animals that did not show the aggression behavior (score < 100) were excluded from the study and the animals that showed the maximum aggression (score ≥ 100) were selected for further study (Tiwari et al., 2010).

##### Scoring of aggression

The Scoring of aggression was done by using the following scale;

0 = Only intermittent nosing.
25 = Showing frequent robust nosing and tail rattling, assuming a fighting position and randomly attacking the intruder.
50 = Tail rattling, screaming, and powerful attacks.
75 = Biting and attacking or striking the intruder for most of the times.
100 = The attacks for the whole duration of observation (Valzelli et al., 1967).

After the induction of aggression by isolation, each group was treated with its respective treatment administered orally daily for 14 consecutive days. On the 14 day, 1 h post-treatment, anti-aggressive activity was observed by introducing the intruder in the cage of each isolated animal.

#### L-DOPA Induced Aggression

The animals were treated with their respective treatment for 14 consecutive days orally. On 14th day, 1 h post-treatment, animals were treated with carbidopa, a peripheral decarboxylase inhibitor (4.28 mg/kg) and levodopa (375mg/kg) orally. Anti-aggressive activity was observed by measuring the biting response (number of bites) by bringing a small rod near the mouth of mice five times, The experiment was conducted three times in half an hour. The mice which bite three of the five times in two observations were considered as aggressive (Yen et al., 1970).

### Analysis of Biochemical Markers

Glutathione, CAT, and SOD were determined as biochemical markers.

#### Preparation of Tissue Homogenate

Animals were anesthetized by using the isoflurane (Cartner et al., 2007). Brain tissues were removed by cervical dislocation of mice and homogenized in Phosphate buffer (pH 7.4) in a ratio of 1/10 (w/v) by using tissue homogenizer. Homogenate was centrifuged at 600 × *g* and +4°C for 10 min. Supernatant was used to analyze the selected biomarkers (GSH, SOD, and CAT).

#### Determination of GSH

Tissue homogenate (1 mL) was precipitated with 10% TCA (1 mL). Four milliliter of phosphate solution and 0.5 mL of DTNB reagent were added to an aliquot of supernatant. Absorbance was measured at 412 nm.

Following formula was used for determination of GSH.

$$\begin{array}{c} \mathrm{GSH}=Y-0.00314÷0.034\times \mathrm{DF}÷\mathrm{BT}\times \mathrm{VU} \end{array}$$

Where, DF = dilution factor which is 1, VU = volume of aliquot, Y = absorbance at 412 nm, BT = tissue homogenate of brain which is 1 mL (Bhangale and Acharya, 2016).

#### Determination of SOD

Pyrogallol solution (0.1 mL) and 0.1 M potassium phosphate buffer (2.8 mL, pH 7.4) were added in tissue homogenate (0.1 mL) to make a reaction mixture of 3 mL. Absorbance was measured at 325 nm (Kim et al., 2016).

Standard curve of SOD was plotted by using different concentrations (10–100 μL) (Saleem et al., 2014).

Following regression equation was used to calculate SOD.

$$\begin{array}{c} Y=0.0095x+0.1939 \end{array}$$

#### Determination of CAT

Tissue homogenate (0.05 mL) was taken. Fifty millimolar phosphate buffer (1.95 mL, pH 7) and 1 mL of 30 mM hydrogen peroxide solution were added in it to make a reaction mixture. Absorbance was measured at 240 nm.

Following formula was used for determination of catalase;

$$\begin{array}{c} \mathrm{CAT}\;\mathrm{activity}\;=\;\;\delta \mathrm{OD}÷E\times \mathrm{vol}.\;\mathrm{of}\;\mathrm{sample}\;(\mathrm{ml})\;\times \;\mathrm{mg}.\;\mathrm{of}\;\mathrm{protein} \end{array}$$

Where, δOD = Changing absorbance/minute

E = Extinction coefficient of hydrogen peroxide having value 0.071 mmol cm^-1^ (Bhangale and Acharya, 2016).

Protein contents were measured by Lowery method and standard curve of protein was plotted at different concentrations of BSA (Hartree, 1972).

Following regression equation was used to determine the protein contents;

$$\begin{array}{c} Y=0.00007571x+0.0000476 \end{array}$$

### Statistical Analysis

All the results were expressed as mean ± standard error of mean (SEM). Two way ANOVA was used to analyze data following Bonferroni *post hoc* test. *P* ≤ 0.001 considered as highly significant, *P* ≤ 0.01 considered as moderately significant, *P* ≤ 0.05 considered as significant with respect to disease control group.

## Results

### Anti-aggressive Activity of Antioxidants against Isolation Induced Aggression

Mice were treated with either ascorbic acid (15.42 and 30.85 mg/kg), beta carotene (1.02 and 2.05 mg/kg), vitamin E (2.5 and 5 mg/kg), and NAC (102.85 and 205.70 mg/kg) to determine the anti-aggressive potential against isolation induced aggression. Diseased control group manifested all the aggression parameters like vigorous noising, tail rattling, squawking, biting for most of the time with maximum score of 100 (**Table 1**).

**Table 1:** Anti-aggressive activity of antioxidants against isolation induced aggression.

Groups	Treatment	Dose (mg/Kg)	Total score
I	Negative control		0 ± 0.00
II	Diseased control	Isolation for one month	100 ± 0.00
III	Standard (Diazepam)	2.5	5.00 ± 5.00
IV	Ascorbic acid	15.42	50.00 ± 17.67
V		30.85	25.00 ± 11.18
VI	Beta carotene	1.02	45.00 ± 14.57
VII		2.05	40.00 ± 20.31
VIII	Vitamin E	2.5	45.00 ± 20.00
IX		5.0	15.00 ± 10.00
X	NAC	102.85	22.00 ± 11.57
XI		205.70	50.00 ± 13.69

*The values are represented as mean ± SEM of ten mins.*

On seventh day of treatment with selected antioxidants, it was observed that low doses of ascorbic acid (15.42 mg/kg), beta carotene (1.02 mg/kg), and vitamin E (2.5 mg/kg) showed dose dependent effect and significantly protected the tail rattling, attacking and biting response with the percentage protection of 50, 55 and 55%, respectively. Surprisingly NAC at low dose (102.85 mg/kg) showed the more protection (78%) against isolation induced aggression than higher dose (205.70%) i.e., 50% protection.

**FIGURE 1:**
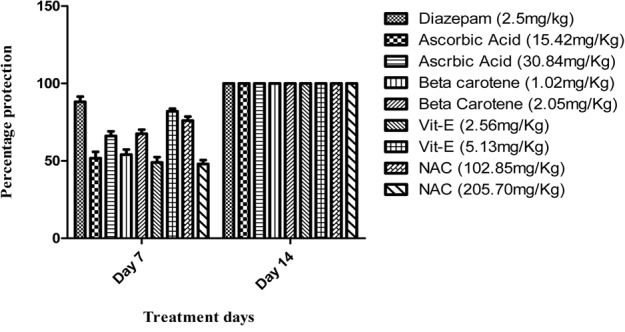
Percentage protection against isolation induced aggression.

On seventh day, vitamin E at high dose (5 mg/kg) significantly (*P* < 0.001) reduced aggressive behavior (85%) as compared to all other treated groups and it was near to diazepam (95%) protection against aggression. All the treated groups significantly (*P* < 0.001) reduced the aggression parameters up to 100% on 14th day of treatment in comparison to diseased control group (**Figure 1**).

### Acute Anti-aggressive Activity against L-DOPA Induced Aggression

Acute protective effect of antioxidants was examined against L-DOPA induced aggression and data is tabulated in **Table 2**.

**Table 2:** Acute anti-aggressive activity of antioxidants against L-DOPA induced aggression.

Groups	Treatment	Dose (mg/Kg)	Aggression parameters after administration of L-DOPA			
			Latency to first bite (Seconds)	Screaming response (No. of screams)	Jumping response (No. of jumps)	Biting response (No. of bites)
I	Negative control		0	0	0	0
II	Standard (diazepam)	2.5	0	0	0	0
III	Diseased control (carbidopa + levodopa)	4.28 + 375	8.6 ± 0.6	1.6 ± 0.4	2.8 ± 0.5	4 ± 0.3
IV	Ascorbic acid	15.42	0	0	0.6 ± 0.4	0
V		30.84	0	0.4 ± 0.4	0	0
VI	Beta carotene	1.02	0	0	0.6 ± 0.6	0
VII		2.05	0	0	0	0
VIII	Vitamin E	2.5	0	0	0	0
IX		5.0	0	0.8 ± 0.8	0	0
X	NAC	102.85	0	0	0	0
XI		205.70	0	0.4 ± 0.4	0	0

*Each value is represented as mean ± SEM.*

A single dose of an antioxidant was administered 30 min before challenging the animals with L-DOPA. The different parameters were observed to score the response. Diseased group exhibited the maximum response to L-DOPA and all the symptoms were shown by this group.

The animals receiving either ascorbic acid (15.42 and 30.85 mg/kg), beta carotene (1.02 and 2.05 mg/kg), vitamin E (2.5 and 5 mg/kg), and NAC (102.85 and 205.70 mg/kg) did not show biting response. However, the ascorbic acid treated group at low dose (15.42 mg/kg) gave jumping response and the same group showed screaming response at high dose (30.84 mg/kg). Jumping response was observed in animals that received beta carotene at low dose (1.02 mg/kg). Only the screaming response was shown by the animals treated with vitamin E (5 mg/kg) and NAC (205.70 mg/kg).

### Chronic Anti-aggressive Activity against L-DOPA Induced Aggression

On day seven, the animals were challenged with L-DOPA, 30 min after receiving daily dose of antioxidants. The animals were observed for half an hour for change in behavior.

It is evident from **Table 3** that all of the pre-treatment protected the animals against L-DOPA induced aggression. Similarly the same animals were given challenged dose of L-DOPA on 14th day, 30 min after receiving daily dose of antioxidants. The results showed that seven consecutive daily doses obliterated the aggression induced by L-DOPA and further dosing for 7 days revealed the same results.

**Table 3:** Chronic anti-aggressive activity of antioxidants against L-DOP induced aggression.

Groups	Treatment	Dose (mg/kg)	Aggression parameters after administration of L-DOPA							
			Latency to first bite (Seconds)		Screaming response (No. of screams)		Jumping response (No. of jumps)		Biting response (No. of bites)	
			On 7^th^ Day	On 14^th^ Day	On 7^th^ Day	On 14^th^ Day	On 7^th^ Day	On 14^th^ Day	On 7^th^ Day	On 14^th^ Day
I	Negative control	NS 0.9% 1ml/kg	0	0	0	0	0	0	0	0
II	Standard (diazepam)	2.5	0	0	0	0	0	0	0	0
III	Diseased control (carbidopa + levodopa)	4.28 + 375	0	0	0	0	0	0	0	0
IV	Ascorbic acid	15.42	0	0	0	0	0	0	0	0
V		30.84	0	0	0	0	0	0	0	0
VI	Beta carotene	1.02	0	0	0	0	0	0	0	0
VII		2.05	0	0	0	0	0	0	0	0
VIII	Vitamin E	2.5	0	0	0	0	0	0	0	0
IX		5.0	0	0	0	0	0	0	0	0
X	NAC	102.85	0	0	0	0	0	0	0	0
XI		205.70	0	0	0	0	0	0	0	0

*Each value is represented as mean ± SEM (n = 10).*

### Estimation of Endogenous Antioxidants in Brain Tissues of Mice Having Isolation Induced Aggression

Endogenous antioxidants such as GSH, SOD, CAT and protein contents are measured in brain tissues to evaluate the effectiveness of selected treatment in socially isolated animals for 1 month. The results are described in **Table 4**.

**Table 4:** Estimation of endogenous antioxidants in brain tissues in mice having isolation induced aggression.

Groups	Treatment	Dose (mg/Kg)	GSH (μg/mg of brain tissue)	SOD (μg/mg of brain tissue)	CAT (μg/mg of brain tissue)
I	Negative control		2.96 ± 0.39	23.16 ± 0.948	5.35 ± 0.07
II	Diseased control	Isolation for 1 month	1.07 ± 0.25	12.78 ± 0.10	1.16 ± 0.01
III	Standard (diazepam)	2.5	5.95 ± 0.02***	31.08 ± 0.24***	4.35 ± 0.07***
IV	Ascorbic acid	15.42	25.10 ± 0.15***	71.07 ± 0.04***	4.63 ± 0.02***
V		30.84	2.18 ± 0.01***	59.67 ± 0.10***	5.07 ± 0.01***
VI	Beta carotene	1.02	17.30 ± 0.204***	68.14 ± 0.59***	6.64 ± 0.01***
VII		2.05	12.35 ± 0.01***	46.44 ± 0.20***	8.56 ± 0.02***
VIII	Vitamin E	2.5	37.36 ± 0.02***	84.34 ± 0.10***	9.75 ± 0.01***
IX		5.0	36.90 ± 0.09***	70.83 ± 0.03***	7.54 ± 0.01***
X	NAC	102.85	25.32 ± 0.16***	91.80 ± 0.03***	2.54 ± 0.01***
XI		205.70	43.37 ± 0.10***	77.02 ± 0.06***	4.71 ± 0.01***

*^∗∗∗^Represents *P* ≤ 0.001 (highly significant) as compared to diseased control.*

*Each value is mean ± SEM (n = 10).*

Results revealed the significant elevated levels of all endogenous antioxidants when compared with the diseased control group. It is evident that there was tremendous increase in SOD and GSH with NAC treated groups at dose of 102.85 and 205.70 mg/kg, respectively. Vitamin E (2.5 mg/kg)-treated group resulted in marked elevation of CAT. Regression equation (Y = 0.0095x + 0.1939) was used to determine the SOD.

### Estimation of Endogenous Antioxidants in Brain Tissues of Mice that Received L-DOPA to Induce Aggression

The brain tissues of mice were analyzed for endogenous antioxidants after challenging them with L-DOPA in chronic anti aggressive protective studies. **Table 5** clearly indicates that there is significant increase in endogenous antioxidants (GSH, SOD, and CAT) after treatment with selected exogenous antioxidants. While beta carotene (2.05 mg/kg)-treated groups exhibited the marked increases in SOD and CAT and ascorbic acid treated mice at high dose (30.84 mg/kg) showed raised level of GSH.

**Table 5:** Estimation of endogenous antioxidants in brain tissues of mice that received L-DOPA to induce aggression.

Groups	Treatment	Dose (mg/ Kg)	GSH (μg/mg of brain tissue)	SOD (μg/mg of brain tissue)	CAT (μg/mg of brain tissue)
I	Negative control		2.96 ± 0.39	23.16 ± 0.94	5.35 ± 0.07
II	Diseases control (carbidopa+ levodopa)	4.28 + 375	1.34 ± 0.01	10.43 ± 0.03	1.151 ± 0.006
III	Standard (diazepam)	2.5	5.95 ± 0.02***	31.08 ± 0.24***	4.35 ± 0.07***
IV	Ascorbic acid	15.42	3.99 ± 0.05***	71.14 ± 0.01***	3.58 ± 0.04***
V		30.84	59.46 ± 0.16***	95.01 ± 0.06***	5.64 ± 0.03***
VI	Beta carotene	1.02	12.56 ± 0.05***	121.5 ± 0.16***	8.31 ± 0.16***
VII		2.05	18.68 ± 0.202***	165.2 ± 0.16***	10.87 ± 0.285***
VIII	Vitamin E	2.5	5.16 ± 0.06***	59.86 ± 0.09***	3.34 ± 0.06***
IX		5.0	7.31 ± 0.03***	70.85 ± 0.06***	7.36 ± 0.04***
X	NAC	102.85	6.35 ± 0.18***	80.56 ± 0.11***	2.63 ± 0.02***
XI		205.70	7.61 ± 0.03***	98.19 ± 0.06***	3.62 ± 0.03***

*^∗∗∗^Represents *P* ≤ 0.001 (highly significant) as compared to diseased control.*

*Each value is mean ± SEM (n = 10).*

## Discussion

Aggression is a major problem worldwide and can be the cause of many crimes. The present study was designed to evaluate the anti-aggression potential of anti-oxidants.

The main source of energy in the mammalian cell is mitochondrial ATP (Cadenas and Davies, 2000; Nicholls, 2005). During the energy transduction, oxygen free radicals are formed which are involved in a variety of diseases such as anxiety, stress, and aggression (Valko et al., 2006). Excessive accumulation of free radicals (ROS) leads to mitochondrial abnormalities, dysfunctioning of neuronal signaling and reduction in neurogenesis (Hovatta et al., 2010). Nitric oxide (NO) claimed as a molecule of the year, by Cech et al. (1992) is generated in tissues via NO synthesis pathway. NO is important in many physiological processes like neurotransmission, defense mechanism and immune regulation, etc. Nitrosative stress is the overall production of RNS (Ridnour et al., 2005). There are different defensive mechanisms in the mammalian cells that are used against oxidative stress like physical defense, preventive, and repair mechanisms (Masella et al., 2005). Enzymes that are used to defend against oxidative stress are SOD, CAT, GSH, etc. (Valko et al., 2006). In present study we analyzed these biochemical markers in aggression. Antioxidants such as ascorbic acid, beta carotene, vitamin E, and NAC were used at two dose levels to evaluate their anti-aggressive potential. Among all, vitamin E at high dose (5 mg/kg) produced greater reduction in aggression (85%) in mice which was comparable to diazepam treatment (95%).

In aggression, generation of oxygen free radicals (i.e., oxidative stress) is increased due to overstimulation of glutamatergic transmission which results in dysfunctioning of neuronal signaling and reduction in neurogenesis (Hovatta et al., 2010). Testosterone level is also increased with oxidative stress which potentiates the aggression that’s why male swiss albino mice were selected for this study (Bhangale and Acharya, 2016). Studies showed that corticosteroids secretion was increased during stress (Krolow et al., 2014) in rodents which is involved in neuronal damage and memory impairment (Esch et al., 2002). Dopamine levels are raised in aggression (Huong et al., 2005; Ryding et al., 2008). Increased glutamatergic transmission caused up-regulation of NMDA receptors that ultimately regulate the dopamine release in striatum (Beiderbeck et al., 2012). Thus, overstimulation of glutamatergic neurons indirectly increased the dopamine levels in aggression (Lang et al., 1995). Whereas, dopamine level in brain was directly increased in L-DOPA induced aggression model. Another study indicated that NMDA receptors antagonists had anti-aggressive behavior due to the blockage of glutamate on these receptors that indirectly reduced the hypersensitivity of D_2_ receptors (Belozertseva and Bespalov, 1999).

It is well established that level of GSH is decreased in socially isolated and L-DOPA induced oxidative damage. Glyoxalase 1 (Glo 1) is a cofactor of GSH that detoxifies various free radicals (ROS). Studies showed that reduction in Glo 1 (controlling gene) causes the anxiety like behavior in various traits of mice that might be due to the impairment of the genetic makeup (Hovatta et al., 2005). Social isolation leads to the generation of free radicals such as superoxide which are ultimately involved in oxidative damage to brain areas. Superoxide is responsible for decreased GSH concentration by different mechanisms (Huong et al., 2005).

Superoxide dismutase concentration was found decreased in aggressive mice which correlate well with aggression and oxidative stress (Garratt and Brooks, 2015). SOD is responsible for catalyzing the highly reactive O_2_^-^ species into the less reactive H_2_O_2_ and oxygen (Bouayed and Bohn, 2010; Dasuri et al., 2013). Biochemical analysis showed the high level of SOD in the treatment groups confirming the neutralization of oxidative species. CAT is present in the cytoplasm and responsible for the conversion of H_2_O_2_ into water and oxygen using the manganese as a co-factor (Dröge, 2002). Enzymatic defense mechanism played an important role in reversing the oxidative stress induced diseases. The biochemical changes associated with aggression are decreased levels of SOD, GSH, and CAT in brain tissues (Hovatta et al., 2005). Our study also supported this phenomenon by showing decreased levels of these anti-oxidant enzymes in aggression induced animal models whereas treatment with antioxidants resulted in anti-aggressive effect by elevating the SOD, GSH, and CAT levels.

It may be concluded that aggression is directly linked with increased dopamine levels in the brain which in turn leads to decrease in SOD, GSH, and CAT levels in brain tissues due to oxidative stress. Ascorbic acid, beta carotene, vitamin E, and N-acetyl cysteine use in aggression could prove beneficial for patients as they prevent the production of free radicals to induce aggression by scavenging them.

## Author Contributions

SH and FA had done the research work, interpreted the results, and critically analyzed the important data for the intellectual content. BA contributed in designing the research protocol. US investigated the integrity of work and provided guidance for publication.

## Conflict of Interest Statement

The authors declare that the research was conducted in the absence of any commercial or financial relationships that could be construed as a potential conflict of interest.
